# No role of IFITM3 in brain tumor formation *in vivo*

**DOI:** 10.18632/oncotarget.13199

**Published:** 2016-11-08

**Authors:** Nevenka Dudvarski Stankovic, Nicola Hoppmann, Marcin Teodorczyk, Ella L. Kim, Matthias Bros, Alf Giese, Frauke Zipp, Mirko H.H. Schmidt

**Affiliations:** ^1^ Molecular Signal Transduction Laboratories, Institute for Microscopic Anatomy and Neurobiology, Focus Program Translational Neuroscience (FTN), Rhine Main Neuroscience Network (rmn^2^), Johannes Gutenberg University, School of Medicine, Mainz, Germany; ^2^ Department of Neurology, Focus Program Translational Neuroscience (FTN) and Research Center for Immunotherapy (FZI), Rhine Main Neuroscience Network (rmn^2^), Johannes Gutenberg University, School of Medicine, Mainz, Germany; ^3^ German Cancer Consortium (DKTK), Heidelberg, Germany; ^4^ German Cancer Research Center (DKFZ), Heidelberg, Germany; ^5^ Translational Oncology Research Group, Department of Neurosurgery, Johannes Gutenberg University, School of Medicine, Mainz, Germany; ^6^ Department of Dermatology, Johannes Gutenberg University, School of Medicine, Mainz, Germany

**Keywords:** brain tumors, cancer stem cells, glioma, IFITM3, irradiation

## Abstract

Glioblastoma multiforme (GBM) is one of the most lethal solid tumors in adults. Despite aggressive treatment approaches for patients, GBM recurrence is inevitable, in part due to the existence of stem-like brain tumor-propagating cells (BTPCs), which produce factors rendering them resistant to radio- and chemotherapy. Comparative transcriptome analysis of irradiated, patient-derived BTPCs revealed a significant upregulation of the interferon-inducible transmembrane protein 3 (IFITM3), suggesting the protein as a factor mediating radio resistance. Previously, IFITM3 has been described to affect glioma cells; therefore, the role of IFITM3 in the formation and progression of brain tumors has been investigated *in vivo*. Intracranial implantation studies using radio-selected BTPCs alongside non-irradiated parental BTPCs in immunodeficient mice displayed no influence of irradiation on animal survival. Furthermore, gain and loss of function studies using BTPCs ectopically expressing IFITM3 or having IFITM3 down-modulated by a shRNA approach, did affect neither tumor growth nor animal survival. Additionally, a syngeneic model based on the mouse glioma cell line GL261 was applied in order to consider the possibility that IFITM3 relies on an intact immune system to unfold its tumorigenic potential. GL261 cells ectopically expressing IFITM3 were implanted into the striatum of immunocompetent mice without influencing the survival of glioma-bearing animals. Lastly, the vasculature and the extent of microglia/macrophage invasion into the tumor were studied in BTPC and GL261 tumors but neither parameter was altered by IFITM3. This report presents for the first time that IFITM3 is upregulated in patient-derived BTPCs upon irradiation but does not affect brain tumor formation or progression *in vivo*.

## INTRODUCTION

Glioblastoma multiforme (GBM) is the most common malignant primary intracranial tumor in adults. It is characterized by widespread invasion throughout the brain, resistance to various therapeutic approaches, and destruction of brain tissue, ultimately leading to death [1]. GBM displays high resistance to chemo- and radiotherapy, thus showing very high recurrence rate and recurrent tumors are even more aggressive [2]. Therefore, the existing standard treatment for GBM, involving maximal surgical resection followed by irradiation and temozolomide (TMZ) chemotherapy, barely prolongs patient median survival from 12.1 to 14.6 months [3]. Such a fast reconstitution of the tumor is in part due to the presence of cancer stem-like cells (glioma stem-like cells [4]) or, more accurately, stem-like brain tumor-propagating cells (BTPCs) [5, 6]. BTPCs are a rare subset of cells within the tumor with significant proliferation capacity and the ability to generate new tumors upon serial transplantation [7, 8]. Data suggest that BTPCs are resistant to conventional radio- and chemotherapy [9] due to the possibility to activate several checkpoint proteins in response to irradiation-induced DNA damage [10]. Hence, BTPCs can survive currently available GBM treatments, efficiently repair damaged DNA and give rise to recurrent tumors [11, 12].

One of the standard glioma therapies, ionizing radiation (IR), has been shown to change the transcriptome in tumors, and genes related to interferon (IFN) pathways are highly upregulated in IR-resistant GBMs [13, 14]. This fact suggested them as factors that might provide gliomas with immune-escape mechanisms. Previously, the human IFN-inducible transmembrane (IFITM) gene family has been identified in a cDNA screen of transcripts upregulated by IFN treatment in a glioblastoma cell line [15]. The IFITM gene family comprises short 2-transmembrane-domain proteins that mediate cellular processes such as cell adhesion, immune cell regulation, germ cell development and bone mineralization [16–22]. Interestingly, head and neck squamous cell carcinomas (HNSCC) are characterized by enhanced expression of IFITM1 [23] with particularly high levels of the protein being expressed at the invasive front of the tumor. Further, IFITM1 has been shown to be suppressed in low-grade astrocytomas, indicating its importance in brain tumors [24]. High expression of IFITM1 induced by IFN-α suppressed proliferation of melanoma cells *in vitro* and in a human xenograft model [25]. Ectopic expression of IFITM3 in melanoma cell lines resulted in the same outcome [26]. IFITM3 has been reported to be upregulated in colon cancer patients as well as in inflamed colon mucosa [27] and was proposed as a diagnostic marker in colorectal tumors [28]. Conversely, knock-down of IFITM3 expression in colon cancer cells by a specific siRNA significantly suppressed proliferation, colony formation, migration, and invasion *in vitro* as well as tumor growth and metastasis in a xenograft model [29]. In line with this finding, the knock-down of both IFITM1 and IFITM3 reduced proliferation, migration, and invasion, and induced cell-cycle arrest and apoptosis in a glioma cell line *in vitro* [30, 31]. These data indicate that IFITM proteins may enhance the malignancy of gliomas. Therefore, the role of IFITM3 in brain tumor initiation and progression has been investigated using various glioma models *in vivo*.

## RESULTS

### Radiation-selected BTPCs displayed an increased expression of IFITM3

**Figure 1:**
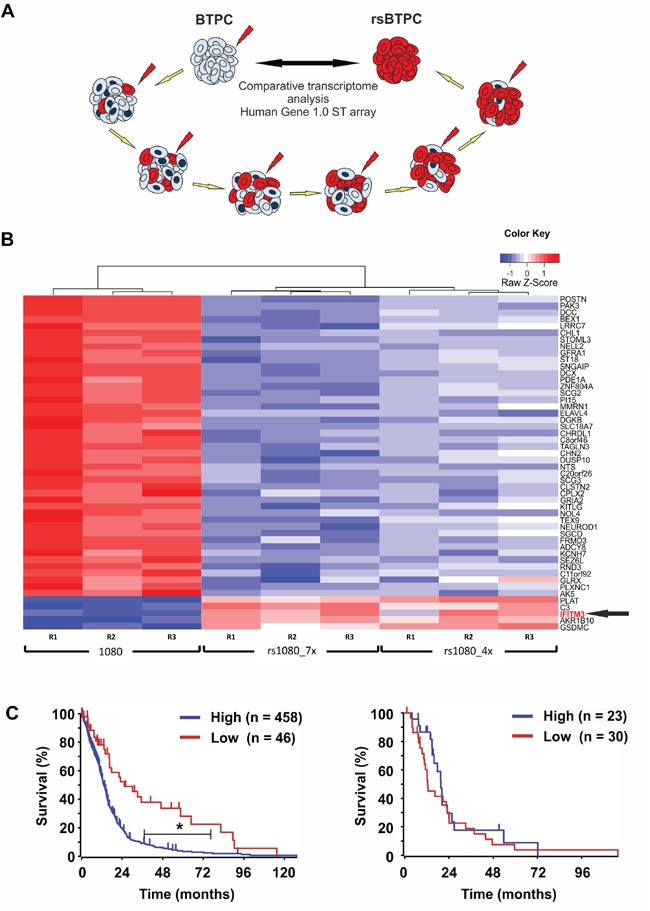
IFITM3 is upregulated in irradiated stem-like brain tumor propagating cells (BTPCs) **A**. BTPC irradiation scheme. **B**. Heat map showing the results obtained from 1080 and its irradiation-selected derivatives rs1080 obtained after irradiation exposure to either seven (“rs1080_7x”) or four (“rs1080_4x”) consecutive rounds of IR. **C**. Overall survival probability of glioblastoma patients based on IFITM3 expression and a TCGA dataset (left panel; IFITM3 high n = 458, IFITM3 low n = 46; *P < 0.001) or in the subset ‘additional irradiation therapy’ (right panel; IFITM3 high n = 23, IFITM3 low n = 30; *P* > 0.05).

**Figure 2:**
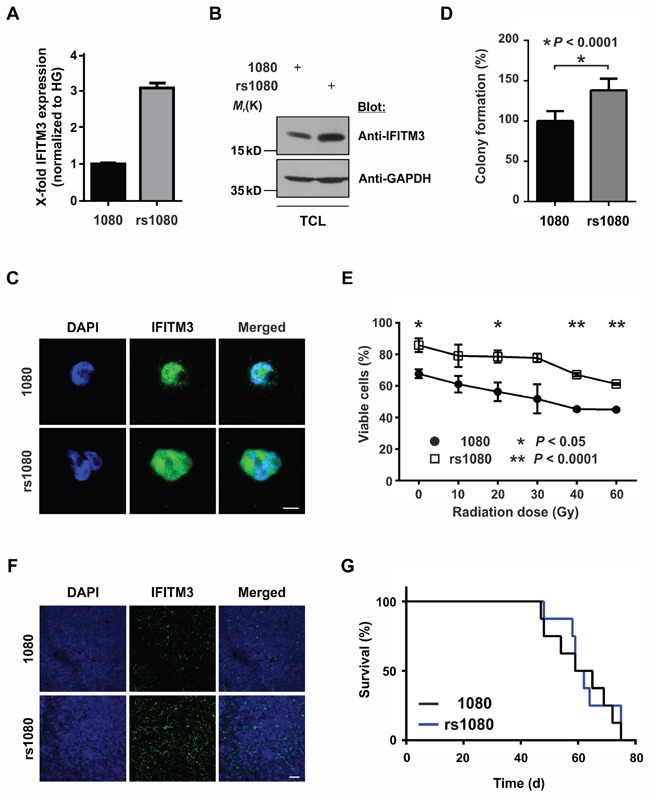
Effects of radio-induced IFITM3 expression on BTPCs **A, B**. and **C**. Fractionized IR of 1080 cells led to an increased expression of IFITM3 **(A)** mRNA and protein as detected by **(B)** Western blot or **(C)** immunocytochemistry. TCL - total cell lysate. Scale bar represents 20 μm. **D**. rs1080 cells displayed enhanced sphere-forming capacity as compared to the parental cell line 1080. **E**. Survival potential of rs1080 and the parental control 1080 48 h after increasing doses of γ-radiation. **F**. Immunostainings showing stabile upregulation of IFITM3 in rs1080 tumors. Scale bar represents 50 μm. **G**. Kaplan-Meier survival curves of mice with rs1080 and control tumors.

In order to identify factors protecting BTPCs from IR, a radio-resistant BTPC line was generated. For this purpose, the line BTPC-1080 (1080) derived from a patient diagnosed with primary GBM WHO°IV was irradiated with 2.5 Gy in seven consecutive passages to select for BTPCs with a radio-resistant phenotype (radio-selected BTPCs, rsBTPCs) in the course of multi-fractionated irradiation *in vitro* (Figure 1A). To explore possible molecular changes induced by irradiation in BTPCs, a comparative transcriptome analysis of 1080 and its radio-selected counterpart rsBTPC-1080 (rs1080) was conducted using the GeneChip Human Gene 1.0 ST array. Immune-signaling proteins were among the most significantly differentially expressed group of genes and IFITM3 was ranked among the top five upregulated genes (Figure 1B). Furthermore, a high expression of IFITM3 was correlated with a poor overall survival of GBM patients according to the Cancer Genome Atlas (TCGA) glioblastoma dataset (Figure 1C, left panel). However, it should be noted that no difference was observed in patients additionally receiving radiotherapy (Figure 1C, right panel). Differential IFITM3 expression in parental and radio-selected BTPCs was further analyzed. Indeed a more than three-fold increase in the expression of IFITM3 was observed in rs1080 as compared to control at mRNA level (Figure 2A) and about two-fold increase at the protein level (Figure 2B+2C).

To evaluate whether rs1080 cells display a survival advantage over non-irradiated parental 1080 cells, 500 cells of each cell line were plated into a 24-well plate. After two weeks, only the spheres with a diameter larger than 50 μm were counted. rs1080 cells yielded a significantly higher number of spheres as compared to their non-irradiated counterparts (Figure 2D; 127.1 ± 5.5% SEM vs 100.0 ± 8.9% SEM; n = 6; *P < 0.05). Furthermore, rs1080 showed higher resistance towards increasing doses of γ-radiation as compared to parental cells (Figure 2E; 85.8 ± 3.9% SEM vs 67.7 ± 2.6% SEM at 0 Gy; 61.3 ± 0.7% SEM vs 45.1 ± 0.5% SEM at 60 Gy; n = 3; *P < 0.05, **P < 0.0001). Data suggest that rs1080 cells acquired a survival benefit and increased tolerance towards IR as compared to parental cells.

In order to evaluate how irradiation and the related radio-induced upregulation of IFITM3 affects tumor formation, 100,000 rs1080 cells were intracranially implanted into the striatum of immunodeficient mice. Subsequently, the overall survival of the animals was monitored. Despite the immunostaining of tumors confirming significantly higher IFITM3 levels in rs1080 as compared to non-irradiated parental cells (Figure 2F), the overall survival was not affected as illustrated by Kaplan-Meier survival curves (Figure 2G; median survival 60.5 vs 62 d in control animals; n = 8; *P* > 0.05).

### Proliferation and radio-resistance of BTPCs upon ectopic IFITM3-myc expression *in vitro*

**Figure 3:**
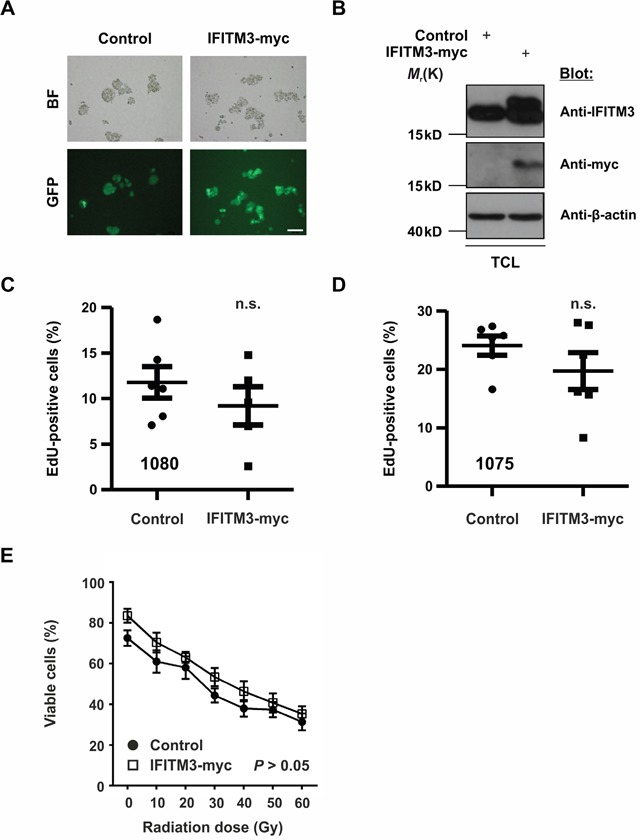
Effects of ectopic IFITM3-myc expression on BTPCs **A**. The expression of IFITM3-myc was verified by the co-expression of the GFP reporter in lentivirus-transduced BTPCs. BF - bright field. Scale bar represents 100 μm. **B**. Western blot analysis of BTPCs, which were transduced with IFITM3-myc, displayed a double band of ectopic IFITM3-myc and endogenous IFITM3 protein. BTPCs, which were transduced with the empty control vector, displayed a band corresponding to endogenous IFITM3 only. TCL - total cell lysate. **C**. and **D**. BTPCs derived from two different patients, (C) 1080 and (D) 1075 cells, were transduced with IFITM3-myc or control and were incubated with EdU for 4 h. EdU incorporation was measured by flow cytometry. Proliferation of cells expressing IFITM3-myc was comparable to those transduced with the negative control. **E**. Survival potential of 1080 IFITM3-myc and control cells, measured 48 h after the application of increasing doses of γ-irradiation. The tendency of 1080 ectopically expressing IFITM3-myc to be more resistant to IR was statistically insignificant.

The upregulation of endogenous IFITM3 upon IR raised the question of the role of this putative radio-resistance marker in BTPCs. The above data suggested that rsBTPCs did not significantly affect the survival of tumor-bearing mice *in vivo*. Therefore, we wondered whether or not the ectopic expression of IFITM3 may affect tumor formation or progression. Thus, as a first step, the influence of IFITM3 on BTPCs was characterized *in vitro*. In order to ectopically express IFITM3 in the absence of IR, IFITM3 cDNA tagged with a myc-tag was inserted into a lentiviral vector, which included GFP as a co-expression reporter. Following the lentiviral transduction of BTPCs with either IFITM3-myc or negative control (GFP only), cells were sorted for GFP expression by a FACS Aria II device (Figure 3A). Western blot verified the ectopic expression of IFITM3-myc protein as compared to control (Figure 3B). Subsequently, 1080 and BTPC-1075 (1075) cells transduced with IFITM3-myc or control, were tested for differences in proliferation using an EdU incorporation assay. After 4 h of incubation, 1080 and 1075 were fixed and stained for quantification by flow cytometry. The results revealed a slight but not statistically significant tendency of IFITM3-myc expressing cells towards decreased proliferation in the case of 1080 (Figure 3C; 11.8 ± 1.7% SEM vs 9.2 ± 2.1% SEM, n = 6; *P* > 0.05) and 1075 (Figure 3D; 24.1 ± 3.1% SEM vs 19.7 ± 3.1% SEM, n = 6; *P* > 0.05). In order to test whether or not the ectopic expression of IFITM3 mediates resistance to irradiation, viability of IFITM3-transduced 1080 cells was assessed once challenged with increasing doses of irradiation. In brief, 20,000 1080 cells transduced with IFITM3-myc or GFP control were exposed to increasing doses of γ-radiation ranging from 10 Gy to 60 Gy, which is the highest dose of IR used during treatment of human gliomas [32]. 48 h after IR, surviving cells were counted and despite 1080 cells ectopically expressing IFITM3-myc displaying a trend of better survival than control, this difference was not statistically significant (Figure 3E; 83.5 ± 3.4% SEM vs 72.5 ± 3.8% SEM at 0 Gy and 35.3 ± 3.8% SEM vs 31.3% ± 4.1% SEM at 60 Gy; n = 3; *P* > 0.05). In summary, the ectopic expression of IFITM3-myc did not cause significant differences in BTPC proliferation or irradiation resistance.

### Characterization of BTPCs upon IFITM3 knock-down *in vitro*

**Figure 4:**
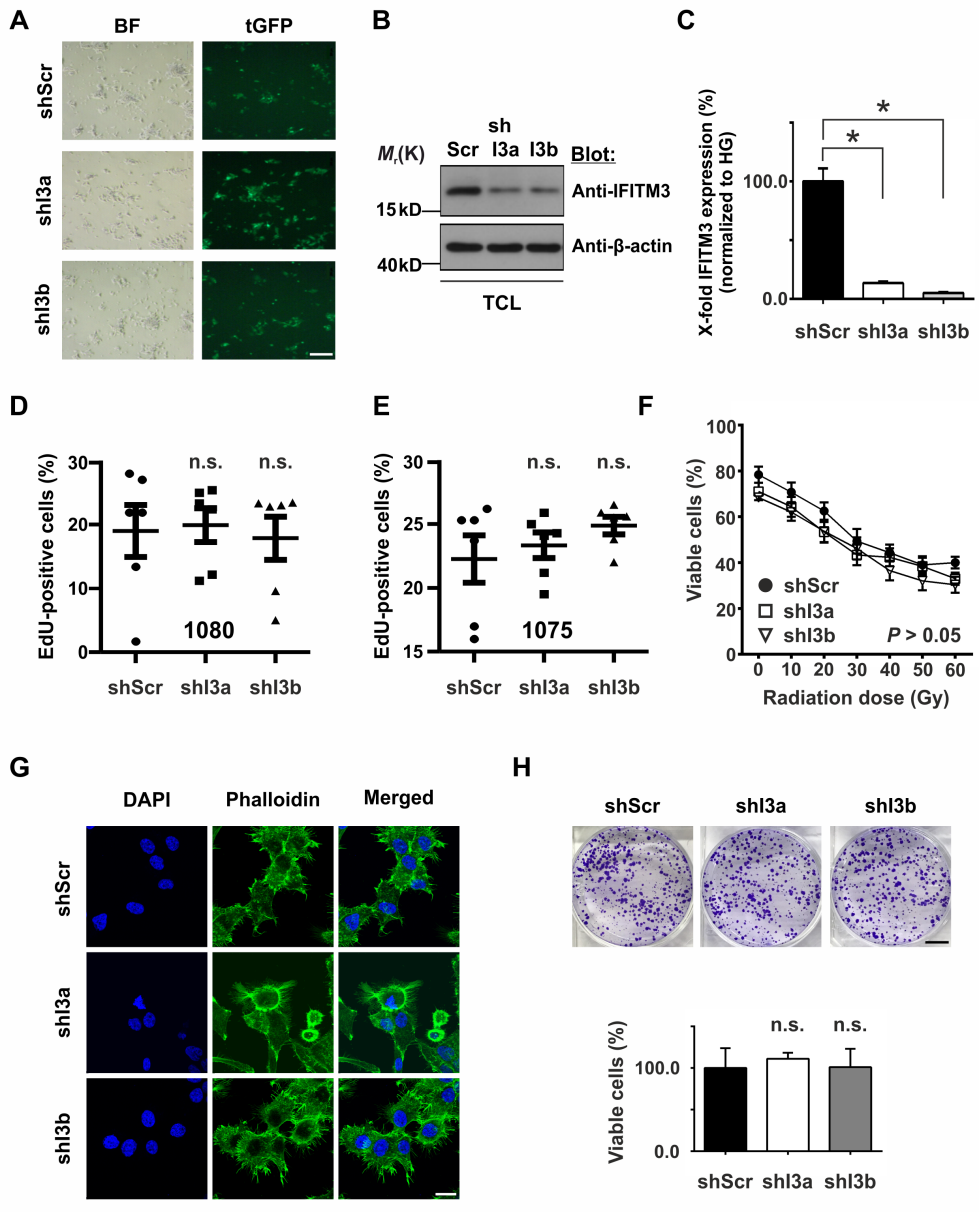
Effects of silencing IFITM3 expression in BTPCs **A**. Expression of shRNAs was verified via the co-expression of tGFP reporter in lentivirus-transduced BTPCs. BF – bright field, tGFP – turboGFP. Scale bar represents 200 μm. **B**. The knock-down of IFITM3 was analyzed via Western blot and **C**. qRT-PCR in BTPCs. shRNA constructs I3a and I3b caused a knock-down of IFITM3 of up to 95%. TCL - total cell lysate. **D**. and **E**. BTPCs derived from two different patients, (D) 1080 and (E) 1075 cells, were transduced with indicated constructs and incubated with EdU for 4 h. EdU incorporation was measured by flow cytometry. Proliferation of cells with a shRNA knock-down of IFITM3 (shI3a, shI3b) was comparable to the respective control cells (shScr); n.s. - not significant. **F**. Survival potential of 1080 cells with silenced IFITM3 expression (shI3a and shI3b) and the control line (shScr), measured 48 h after the application of increasing doses of γ-irradiation. The tendency of shI3a and shI3b to be more sensitive to IR was statistically insignificant. **G**. 1080 shI3a, shI3b and shScr showing no difference in the distribution or intensity of F-actin labeled by Phalloidin staining. Scale bar represents 20 μm. **H**. Comparison of the *in vitro* clonogenecity of 1080 cells transduced with anti-IFITM3 shRNAs (shI3a and shI3b) or negative control (shScr). The intensity of crystal violet stainings was assessed according to optical density at 570 nm; n.s. - not significant. Scale bar represents 7 mm.

In light of the above findings we wondered if the reduction of IFITM3 may affect the functional characteristics of BTPCs. In order to knock-down endogenous IFITM3 in BTPCs, anti-IFITM3 shRNAs (shI3a and 3b) or a scrambled control (shScr) were inserted into a lentiviral vector and linked to turboGFP (tGFP) and puromycin expression via an IRES sequence. Upon lentiviral transduction and puromycin selection of BTPCs (Figure 4A), the knock-down of IFITM3 protein was confirmed by Western blot (Figure 4B) and quantitative reverse transcriptase-polymerase chain reaction (qRT-PCR) (Figure 4C). Anti-IFITM3 shRNA clones I3a and I3b caused the strongest IFITM3-knock-down of about 70% on the protein level (Figure 4B) and up to 95% on the mRNA level (Figure 4C; 13.4 ± 1.3% SEM (shI3a) or 5.1 ± 0.6% SEM (shI3b) vs 100.0 ± 11.0% SEM (shScr); n = 3; *P < 0.0001). BTPCs with silenced expression of IFITM3 proliferated equally fast as compared to control in the case of 1080 cells (Figure 4D; 19.0 ± 4.1% SEM (shI3a) or 20.0 ± 2.6% SEM (shI3b) vs 17.9 ± 3.4% SEM (shScr); n = 6; *P* > 0.05) and 1075 cells (Figure 4E; 22.3 ± 1.9% SEM (shI3a) or 23.3 ± 1.0% SEM (shI3b) vs 24.9 ± 0.7% SEM (shScr); n = 6; *P* > 0.05). Although the knock-down of IFITM3 rendered 1080 cells slightly more sensitive to IR as compared to control, the difference remained statistically insignificant (Figure 4F; 71.0 ± 3.8% SEM (shI3a) or 68.3 ± 1.8% SEM (shI3b) vs 78.3 ± 3.5% SEM (shScr); n = 3; *P* > 0.05 at 0 Gy and 33.0 ± 2.6% SEM (shI3a) or 30.3 ± 3.5% SEM (shI3b) vs 40.0 ± 2.5% SEM (shScr); n = 3; *P* > 0.05 at 60 Gy).

Furthermore, we questioned if silenced IFITM3 expression in 1080 cells caused cytoskeletal alterations. In order to address this, 60,000 shI3a, shI3b or shScr 1080 cells were plated on ornithine-coated coverslips 3 d prior to staining. Phalloidin staining revealed a predominant association of F-actin with the cell membrane and numerous lamellipodia in all samples (Figure 4G). However, no recognizable difference in the intensity or distribution of F-actin was observed among 1080 cells transduced with shI3a or shI3b and the shScr control.

In order to analyze whether or not IFITM3 influenced the malignancy of 1080 cells treated with shI3a, shI3b and shScr, clonogenicity assays were performed. 2,000 cells were seeded per dish and were grown at low density for 14 d. Subsequently, the colonies that had formed were stained with crystal violet, lysed and analyzed in a spectrophotometer. 1080 cells transduced with anti-IFITM3 shRNAs formed colonies similar in number and size as the control 1080 shScr (Figure 4H; 111.0 ± 7.2% SEM (shI3a) or 101.0 ± 22.1% SEM (shI3b) vs 100.0 ± 23.7% SEM (shScr); n = 3; *P* > 0.05). In conclusion, no difference in the clonogenic potential was observed among the three lines, suggesting that IFITM3 did not play a significant role in BTPC tumorigenicity *in vitro*. In sum, the down-modulation of IFITM3 did not affect proliferation, radio-resistance, cytoskeletal organization or colony formation of BTPCs *in vitro*.

### Functional analysis of ectopic expression of IFITM3 *in vivo*

**Figure 5:**
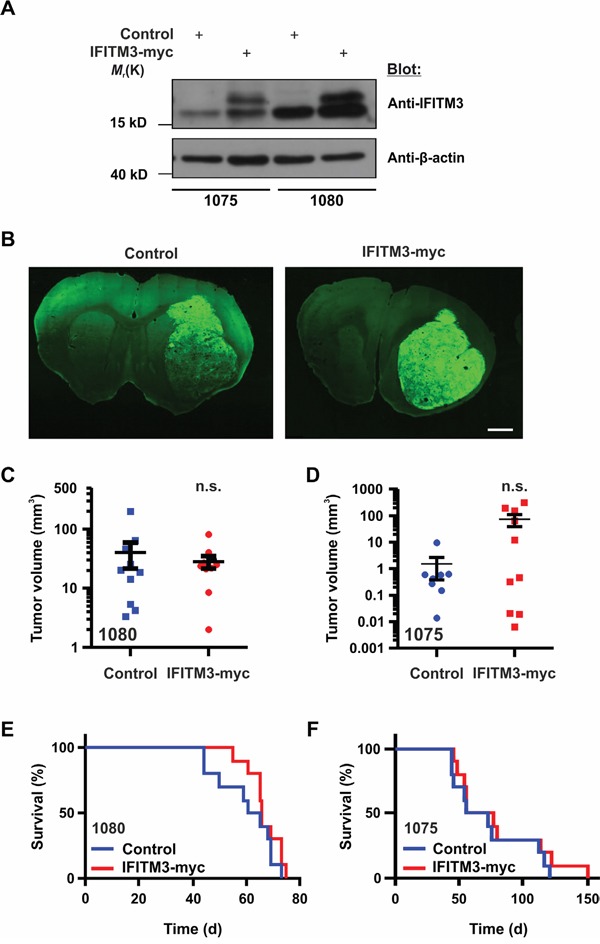
Ectopic expression of IFITM3-myc in BTPCs does not affect tumor growth in vivo **A**. Western blot analysis of BTPCs transduced with the IFITM3-myc-encoding vector (the upper band represents ectopic IFITM3-myc). **B**. Coronal brain slices of GFP reporter-expressing tumors consisting of control cells (left panel) or cells expressing ectopic IFITM3-myc (right panel). The tumor area of at least 10 slices per brain tumor was measured using ImageJ software. Scale bar represents 0.5 cm. **C**. and **D**. Measurement of tumor volumes after 7 weeks incubation time after ecoptic IFITM3-myc expression using **(C)** 1080 and **(D)** 1075 cells. **E**. and **F**. Kaplan-Meier survival curves of mice harboring ectopic IFITM3-myc-expressing tumors using **(E)** 1080 and **(F)** 1075 cells.

Following these *in vitro* proliferation assays, the effect of enhanced IFITM3 expression on tumor growth was analyzed in mouse xenograft tumor models. For this purpose, BTPCs ectopically expressing IFITM3-myc or control were generated and sorted by FACS as described above. A pool of 100,000 IFITM3-myc or GFP-transduced BTPCs were intracranially implanted into immunodeficient NMRI nude mice (Figure 5A). Seven weeks after implantation, mice were sacrificed and tumor volumes were measured (Figure 5B). Although all mice developed tumors, intragroup variations in tumor volumes between 0.2 and 300 mm^3^ were observed. Both experimental groups exhibited similar distributions and no significant differences in tumor size were observed (Figure 5C; 25.2 ± 6.8 mm^3^ SEM vs 40.3 ± 18.8 mm^3^ SEM in control animals; n = 10; *P* > 0.05; Figure 5D; 64.9 ± 34.7 mm^3^ SEM vs 1.5 ± 1.1 mm^3^ SEM in control animals; n ≥ 8; *P* > 0.05). Further, the overall survival of the mice orthotopically implanted with the respective tumor cells as described above was monitored but the ectopic expression of IFITM3-myc did not change the survival of mice transplanted with 1080 cells (Figure 5E; median survival 63 vs 66 d in control animals; n = 10; *P* > 0.05) or 1075 cells (Figure 5F; median survival 64 vs 66 d in control animals; n = 10; *P* > 0.05).

### Influence of IFITM3 on microglial recruitment and glioma angiogenesis

**Figure 6:**
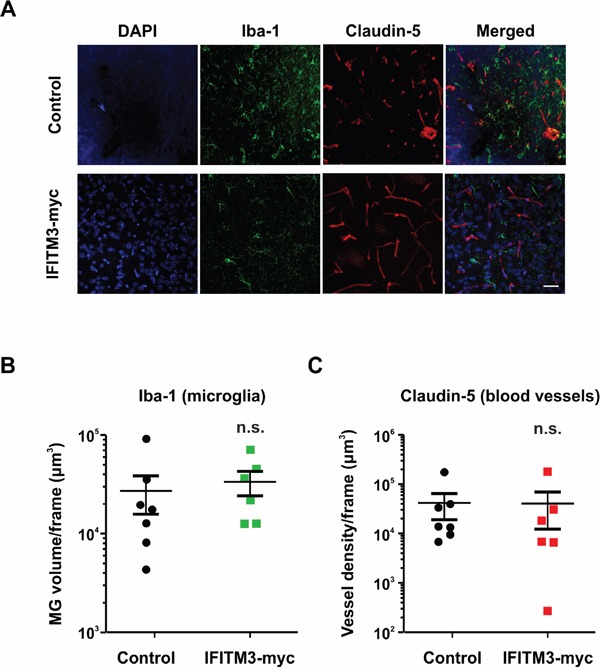
Ectopic expression of IFITM3 in BTPCs has no significant effect on microglia recruitment or neoangiogenesis in tumors **A**. A representative image of Iba-1 (microglia marker) and Claudin-5 (blood vessel marker) staining of BTPC-tumors. Scale bar represents 50 μm. **B**. The microglia volume of at least 3 slices per brain tumor was quantified with Imaris software (n ≥ 6; no significant differences between experimental groups according to Mann-Whitney *U* - test). **C**. The blood vessel density of at least 3 slices per brain tumor was quantified with Imaris software (n ≥ 6; no significant differences between experimental groups according to Mann-Whitney *U* - test).

As IFITM proteins play a role in immune cell regulation, the ectopic expression of IFITM3 may influence microglia/macrophages (MG) infiltration and blood vessel formation in BTPC-derived tumors. MG were quantified by the staining of brain tumor slices for ionized calcium-binding adapter molecule-1 (Iba-1) (Figure 6A). Quantification of fluorescence stainings by Imaris software revealed no significant differences between the ectopic IFITM3-myc expressing and control group, suggesting that both experimental groups have been infiltrated by MG to a similar extent (Figure 6B).

Furthermore, tumors larger than 2 mm^3^ are unlikely to survive without the proper vasculature and thus upregulate pro-angiogenic signaling factors. Therefore, it was investigated whether or not the tumor vasculature was altered in BTPC xenografts upon ectopic IFITM3 expression. The vessels were visualized by staining the brain sections for claudin-5 (Figure 6A). Quantification of the blood vessel volume using the Imaris software did not reveal any gross differences between BTPC tumors formed in the presence of ectopic IFITM3-myc or control (Figure 6C). Data suggest that tumor neoangiogenesis in BTPC xenografts was not affected by the ectopic expression of IFITM3-myc.

### Functional analysis of IFITM3 knock-down *in vivo*

**Figure 7:**
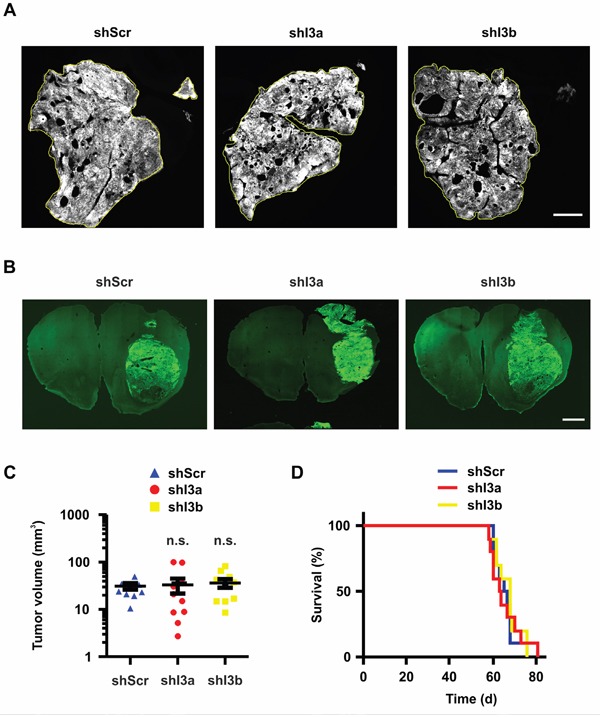
Knock-down of IFITM3 in BTPCs has no significant effect on tumor growth in vivo **A**. Coronal brain slices of tGFP-expressing tumors consisting of control (left panel) and IFITM3 knock-down cells (right panels). The tumor area of at least 10 slices per brain tumor was measured using ImageJ software. Scale bar represents 1 cm. **B**. Coronal brain slices of whole mount brains bearing tGFP-expressing 1080 tumors consisting of control (left panel) and IFITM3 knock-down cells (right panels). Scale bar represents 0.5 cm. **C**. Measurement of tumor volumes after 7 weeks incubation time. The tumor area of at least 10 slices per brain tumor was measured using ImageJ software. **D**. The survival of mice harboring IFITM3 knock-down in 1080 vs non-targeting control. Both tumor volume and survival were not statistically different between the groups.

Putatively, ectopic IFITM3-myc expression did not affect tumor cell properties to a significant extent as 1080 cells already expressed a significant amount of endogenous IFITM3. Therefore, its downregulation was investigated in greater detail. For this purpose, 100,000 BTPCs were transduced with anti-IFITM3 shRNAs or a non-targeting shRNA construct and were intracranially implanted into immuno-deficient NMRI nude mice. Subsequently, the same procedure was followed as for ectopic IFITM3-myc expressing BTPCs. Tumor volume estimation was based on tGFP expression (Figure 7A+7B) and ranged between 2 and 100 mm^3^ (Figure 7C), similar in size as the tumors ectopically expressing IFITM3-myc. Moreover, there was no significant difference between the experimental groups (Figure 7C; 31.0 ± 5.0 mm^3^ SEM (shI3a) or 33.0 ± 11.5 mm^3^ SEM (shI3b) vs 36.2 ± 7.6 mm^3^ SEM (shScr); n = 10; *P* > 0.05 according to one-way ANOVA). The overall survival, as illustrated by Kaplan-Meier survival curves, was not altered (Figure 7D; median survival 63.5 (shI3a) or 68 (shI3b) vs 66 d (shScr); n = 10; *P* > 0.05). Furthermore, the median survival of these animals was comparable to mice bearing tumors expressing radio-induced IFITM3 (Figure 2G) or ectopic IFITM3-myc (Figure 5E+5F). Based on *in vivo* data presented above, modulation of IFITM3 did not affect tumor growth and progression in BTPC xenografts.

### Functional analysis of ectopic IFITM3 expression in a syngeneic mouse tumor model

**Figure 8:**
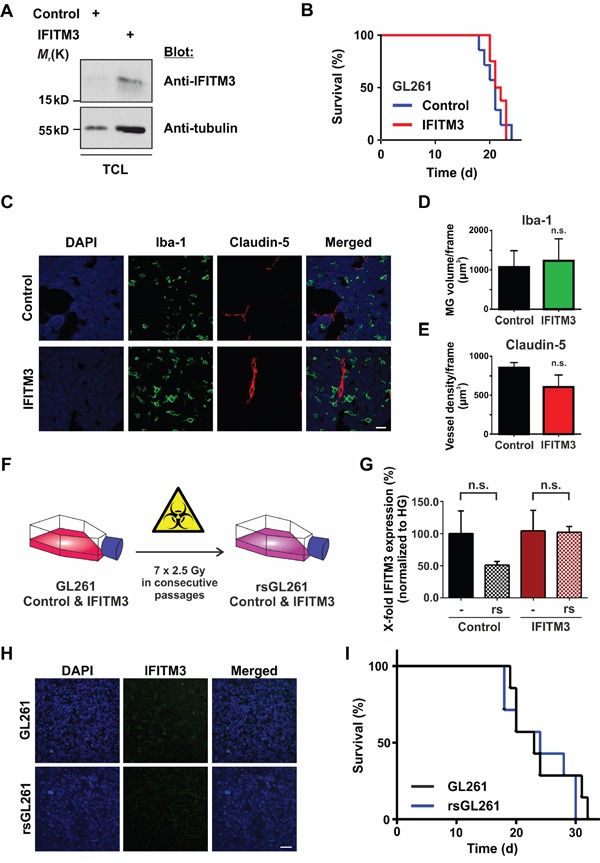
No effect of IFITM3 in a syngeneic GL261 glioma model **A**. Western blot analysis of GL261 cells that were transduced with the IFITM3-encoding vector confirms ectopic expression of IFITM3. TCL - total cell lysate. **B**. The survival of mice harboring GL261 tumors ectopically expressing IFITM3-myc was comparable to dsRed-expressing negative controls. **C**. A representative image of Iba-1 (microglia marker) and Claudin 5 (blood vessel marker) staining of GL261 tumors expressing IFITM3-myc or negative control. Scale bar represents 30 μm. **D**. The volume of MG of at least 5 slices per brain tumor was quantified with Imaris software (n = 3; n. s. - no significant differences between experimental groups according to Mann-Whitney *U* - test). **E**. Blood vessel density of at least 5 slices per brain tumor was quantified with Imaris software (n = 3; n. s. - no significant differences between experimental groups according to Mann-Whitney *U* - test). **F**. GL261 cells ectopically expressing IFITM3-myc or the control cell line were exposed to 7 consecutive rounds of γ-irradiation. **G**. IR of GL261 did not induce the expression of endogenous IFITM3, n.s. - not significant. **H**. IFITM3 immunostainings show that GL261 tumors did not express endogenous IFITM3 upon IR. Scale bar represents 50 μm. **I**. Kaplan-Meier survival curves of mice bearing GL261 tumors show no difference upon the transplantation of radio-selected cells (rsGL261) or parental controls (GL261).

Since IFN signaling affects many aspects of the immune system, we utilized a syngeneic tumor model in order to test the impact of ectopic IFITM3 expression in a functional immune environment. Therefore, the mouse glioma cell line GL261 was transduced with a lentivirus encoding for IFITM3-IRES-tdTomato or a control lentivirus encoding for dsRed-IRES-tdTomato. Upon FACS Aria II cell sorting and based on the strength of tdTomato signal, the ectopic expression of IFITM3 was verified by Western blot (Figure 8A). 100,000 IFITM3- or dsRed-expressing GL261 cells were intracranially implanted into the striatum of adult, male, immunocompetent mice. As in the BTPC mouse model, no difference was observed in the survival of implanted animals (Figure 8B; median survival 21 vs 21.5 d; n = 8; *P* > 0.05). Furthermore, the staining for Iba-1 and Claudin-5 in the GL261 tumors did not yield obvious differences among the experimental groups (Figure 8C - 8E). Thus, the ectopic expression of IFITM3 did not affect angiogenesis or MG recruitment in the immune competent environment of the GL261 glioma model.

Moreover, we wondered if IR induced IFITM3 expression in GL261 cells in a similar manner as it did in BTPCs. Therefore, we exposed GL261 ectopically expressing human IFITM3 or dsRed (control) to seven consecutive rounds of irradiation (Figure 8F) and performed qRT-PCR analysis using mouse-specific IFITM3 primers. Results revealed that GL261 cells did not express IFITM3 (Ct values ≥ 36) upon this treatment (Figure 8G; 51.0 ± 5.8% SEM (rsGL261) vs 100.0 ± 35.3% SEM (GL261) or 102.0 ± 9.1% SEM (rsIFITM3) vs 104.3 ± 31.9% SEM (IFITM3); n = 3; *P* > 0.05). Subsequently, 100,000 rsGL261 cells or their non-irradiated counterparts were intracranially implanted into the striatum of C57BL/6 mice. Immunostainings of tumor biopsies confirmed that radio-selection did not induce the expression of IFITM3 in GL261 cells (Figure 8H). As was the case with the xenografts analyzed above, no difference was observed in the survival of animals implanted with rsGL261 or GL261 (Figure 8I, median survival 24 vs 23 d in control animals; n = 7; *P* > 0.05).

## DISCUSSION

There is no clear consensus in the literature on the contribution of IFITM proteins to tumor growth. In contradiction, both increased and decreased expression of these proteins is described in different types of cancer [23, 27, 28]. Further, there are data showing that IFITM expression correlates with enhanced proliferation of cancer cells, but it is also linked to reduced proliferation rate [25, 26, 29–31]. TCGA-based analysis of glioblastoma patient survival indicated a highly significant correlation between IFITM3 expression and diminished survival. However, this data should be interpreted cautiously as this difference is not observed in patients receiving additional radiotherapy. Moreover, according to another database, namely REMBRANDT [33], there is very low variation in IFITM3 expression in glioma samples (data not shown). In recent studies, IFITM1 and IFITM3 were proposed as protumorigenic factors, as knock-down of these proteins inhibited proliferation, migration, invasion and colony formation of glioma cell lines [30, 31]. These experiments, however, were conducted only *in vitro* and the role of IFITM3 in the glioma formation and progression has not been addressed in a mouse tumor model, which this study sought to resolve.

In order to evaluate the effects of IFITM3 on glioma growth *in vivo*, rsBTPCs with an enhanced expression of IFITM3 were used in glioma xenografts. Although all animals developed tumors, the overall survival was not altered. However, the upregulation of IFITM3 achieved by fractionated IR was maybe not sufficient to unravel IFITM3′s full oncogenic potential. Therefore, IFITM3 was ectopically expressed in BTPCs. Measurement of EdU incorporation revealed that the ectopic expression of IFITM3 did not significantly alter the proliferation rate of BTPCs. Furthermore, the orthotopic implantation of lentiviral-transduced BTPCs displaying enhanced IFITM3 expression into immunocompromised mice gave rise to tumors in all animals but the tumors in the experimental and control group were similar in size. Along this line, the overall survival of tumor-bearing mice was not altered by the ectopic expression of IFITM3. Subsequently, the study was extended to the examination of the distribution of MG and blood vessels within the tumors. However, the ectopic expression of IFITM3 affected none of these parameters either.

As the diminished expression of IFITM3 has already been reported to have a critical role in tumor growth [29], a stable knock-down of IFITM3 in BTPCs has been achieved. Proliferation was measured by EdU incorporation *in vitro* as described above but was not significantly altered in BTPCs with reduced IFITM3 expression. Furthermore, the knock-down of IFITM3 in 1080 cells neither affected the actin cytoskeleton as detected by Phalloidin staining nor the formation of clones in a clonogenic assay as assessed by crystal violet stainings *in vitro*. Moreover, the intracranial implantation of BTPCs transduced with lentiviruses encoding for IFITM3-specific shRNAs or shScr control into immunocompromised mice gave rise to tumors of comparable sizes and did not alter the overall survival of tumor-bearing mice. Again, the silenced expression of IFITM3 did alter neither MG infiltration into the tumor nor blood vessel neoangiogenesis. In addition, the median survival of animals bearing tumors with reduced levels of IFITM3 was not different from the ones ectopically expressing IFITM3 or showing an irradiation-induced upregulation of IFITM3. Data suggest IFITM3 did neither affect tumor growth in a BTPC mouse xenograft nor render the cells resistant to IR.

As of yet, a reduction of the proliferation in conventional glioma cell lines as a consequence of IFITM3 knock-down has not been confirmed in BTPCs. It is possible that IFITM3 does not have a significant role in proliferation of BTPCs in contrary to established cell lines. Alternatively, the modulation of IFITM3 expression may activate compensatory mechanisms carried by highly similar homologs IFITM1 or IFITM2 [34]. Along these lines, *Ifitm3*^-/-^ mice have no discernable phenotype [35] with the exception of succumbing to sub-lethal doses of influenza virus [36, 37]. Yet, another possibility is that the effect of IFITM3 on BTPC proliferation may be confined to certain periods of time after exposure to irradiation. Finally, the conflicting findings of pro- and anti-tumorigenic roles of IFITM proteins in different tumors might depend on available interaction partners and signals from the specific tumor microenvironment [31].

Complementing the BTPC experiments, IFITM3 was expressed in a mouse cell line GL261 transplanted into immunocompetent animals in order to test the hypothesis proposed by Yu et al. [30]. The authors suggest that IFITM1, a homolog of IFITM3, may be produced by glioma cells so as to antagonize the attack by the host immune system by promoting cell proliferation and invasion. The obtained results were in line with the data acquired from human BTPCs as no significant difference in survival or functional characteristics of tumors between experimental groups was observed.

IFN-related genes, and IFITM proteins in particular, have been reported to be upregulated after the IR of cell lines derived from prostate [38], glioma, breast [14] and leukemic tumors [39]. However, IFN-β did not contribute to radiosensitization of human GBM cell lines [40] or BTPCs [41]. This data is in line with our findings as neither knock-down nor ectopic expression of IFITM3 changed BTPC radio-resistance. Clinical research provides valuable data on IFN signaling in brain tumors. Intratumoral treatment of gliomas with recombinant IFN-γ did not improve the survival of patients receiving 60 Gy of IR [42]. On the other hand, the combination of IFNs and chemotherapy appeared to be more effective as administration of IFN-β was beneficial for patients as compared to TMZ treatment alone [43]. An experiment with U87 xenografts suggests that IFN-β should precede IR as IFN-β disrupted the vascular niche of glioma stem cells in transplanted SCID mice [44]. Though one study reported a benefit of the combination of IFN-α plus IR [45], other clinical trials produced opposite results. Addition of IFN-α to IR plus carmustine chemotherapy did not improve the survival of newly-diagnosed high-grade glioma patients [46]. Moreover, the combination of IFN-α and 13-cis-retinoic acid enhanced glioma susceptibility to IR *in vitro* but did not result in a better outcome in patients treated with IR plus IFN-α as compared to IR alone [47].

All in all, our data reveal that IFITM3 has only a marginal, if any, oncogenic potential when examined in human primary cells or in various animal models. Although no significant effects have been observed in our paradigms, this does not dismiss the possibility that including more variables, e.g., performing IR *in vivo*, would uncover a more subtle outcome of manipulating IFITM3 expression. Nevertheless, the role of IFITM3 in glioma formation *in vivo* has not been investigated, yet. At this stage, data presented in this study argues against a significant role of IFITM3 in glioma formation, progression or radio-resistance.

## MATERIALS AND METHODS

### Cell isolation and culture methods

Human glioma 1080 and 1075 cells were isolated from fresh glioblastoma biopsies as described elsewhere [48, 49]. Briefly, tumor biopsies were dissociated with the Neural Dissociation Kit (Miltenyi Biotec GmbH, Bergisch Gladbach, Germany). 1080 and 1075 were derived from different patients both diagnosed with primary GBM WHO°IV and operated at the Department of Neurosurgery of the University Medical Centre Göttingen. Human brain tumor-propagating cells were grown under sterile conditions in Neurobasal-A Medium (Gibco – Thermo Fischer Scientific, Waltham, Massachusetts, USA), supplemented with B27-Supplement (Invitrogen, Carlsbad, California, USA), 0.1% Bovine serum albumin (BSA) (SERVA Electrophoresis GmbH, Heidelberg, Germany), Penicillin/Streptomycin (Gibco), and the growths factors bFGF and EGF (20 ng/ml each) (Peprotech, Rocky Hill, Connecticut, USA) to select for stem and progenitor cells. To split BTPC spheres, cells were treated with Accutase (PAA, Egelsbach, Germany) at 37°C for 10 min. 400,000 - 500,000 cells were plated into a 10 cm^2^ petri dish or T75 culture flask and stored in an incubator at 37°C, 5% CO_2_.

The patient's written informed consent for using excessive tumor tissue for research purposes was obtained. The use of tumor tissue was approved by the Institutional Review Board of the University Medical Centre Göttingen.

### Selection of radio-resistant BTPCs

Gliomaspheres were triturated to a single cell level by treatment with Trypsin (PAA) and counted. 100,000 - 250,000 cells were plated in 5 ml of medium in T25 flasks and subjected to 2.5 Gy of IR at a dose rate of 1.0 Gy/min. Radiated cells were cultured until new gliomaspheres formed. This procedure was repeated for seven consecutive passages.

### Selection of radio-resistant GL261

GL261-IFITM3-tdTomato and negative control GL261-tdTomato were triturated to a single cell level by treatment with Trypsin and counted. 500,000 cells were subjected to 2.5 Gy of IR at a dose rate of 1.0 Gy/min. Radiated cells were cultured till at least 80% confluency. This procedure was repeated for seven consecutive passages.

### EdU proliferation assay

Proliferation of cells was measured by EdU incorporation over 3 h and 6 h of treatment and staining as well as analysis was pursued as described in the Click-iT^®^ EdU Flow Cytometry Assay Kit's (Thermo Fischer Scientific) manual.

Briefly, the cells were brought to a single cell level by Accutase treatment, counted and seeded into a 6-well plate at 100,000 cells per 2 ml culture medium per well. After 4 d incubation and colony formation time, 2 μM EdU was added to according wells in duplicates for the desired amount of time. The cells were brought to a single cell level, washed with 1%BSA/PBS and re-suspended in either Component D fixative or 2% PFA/PBS at room temperature (RT) for 15 min. Fixative was washed off by addition of 1% BSA/PBS and the pellet was incubated in Saponin-based permeabilization and wash reagent (Component E) at RT for 15 min. Afterwards cells were stained with a mixture consisting of PBS, CuSO4, anti-EdU-Alexa Fluor 488 or anti-EdU-Alexa Fluor 647 at RT for 30 min. Upon washing and staining with 1 μg/ml DAPI, samples were analyzed by FACS Canto II (BD Biosciences, San Jose, California, USA).

### Cloning of IFITM3

A Glycerol Stock of bacteria expressing IFITM3 clone with the accession number BC070243.1 [Homo sapiens interferon induced transmembrane protein 3 (1-8U), mRNA (cDNA clone MGC:88228 IMAGE:30401041), complete cds], was purchased from BioSource (Thermo Fischer Scientific). IFITM3 was cloned from the bacterial stock by colony PCR with forward primer 5’ - AAAATCTAGAATGAATCACAC TGTCCAAA - 3’ and reverse primer 5’ – AAAAGGATCCCTACAGATCTTCTTCAGAAATAAGTTTTTGTTC CTCGAG TCCATAGGCCTGGAAGAT - 3’ in a thermo cycler. The mammalian expression vector pCDH-Ef1a-MCS-IRES-copGFP was purchased from Biocat (Heidelberg, Germany). Chemically competent E.coli TOP10 bacteria (Invitrogen) transformed with the vector, were selected with Ampicillin. For vector amplification, colony was grown in LB-Medium and plasmids were isolated in a maxi preparation according to protocol (QIAGEN® Plasmid Purification Kit, Qiagen, Hilden, Germany). Upon digestion of both vector and insert with XbaI and BamHI, they were purified (QIAquick Gel Extraction Kit, Qiagen) from 1% agarose gel. The ligation product was transformed into E.coli TOP10 bacteria and grown on agar selection plates with Ampicillin. Five colonies were picked and grown in LB-Medium for mini plasmid preparation according to protocol (Plasmid Mini Preparation Kit, PEQLAB, Erlangen, Germany). Clones were checked with restriction enzyme digestions and confirmed with sequencing (StarSEQ, Mainz, Germany).

### Lentiviral production and transduction of cells

Lentivirus was generated as described previously [50]. 300,000 BTPCs were seeded onto ornithine-coated (Sigma-Aldrich, St. Louis, Missouri, USA) 6-well plates and infected with the virus in the presence of polybrene. 48 h after infection, cells were transferred to S1 area and successful transduction was secured by visualization of GFP or tGFP expression. Regarding transduction of GL261 cells, 500,000 cells were seeded in a well of a 6-well plate a day prior to infection. Upon addition of virus with the fresh medium, plates were centrifuged at 625 g, 31°C for 1 h. Successful transduction was secured by visualization of tdTomato expression.

### SDS-PAGE and western blot

To prepare protein samples for gel electrophoresis, 10 - 30 μg of total protein lysate was mixed with 5 x Laemmli buffer and H_2_O for equal sample amounts and the proteins were denatured at 95°C for 5 min. Proteins were separated on 10 - 15% gels under reducing conditions at constant 80 - 100 V, max mA for 2 h. Proteins were then transferred onto nitrocellulose membranes (GE Watter & Process, Herentals, Belgium) by wet Western blotting at 100 V, max mA for 1.5 h. Unspecific binding sites were blocked with TBS-T + 5% skim milk powder at RT for 60 min on a shaker. Membranes were incubated with indicated primary antibodies in TBS-T + 5% BSA, 0.1% sodium azide at 4°C overnight. The next day, membranes were washed 3 x in TBS-T and probed with the corresponding HRP-conjugated secondary antibodies in TBS-T + 3% BSA at RT for 1.5 h. The HRP signal was detected by incubation of the membrane with advanced ECL solution and consecutive exposure to X-ray films.

### Clonogenic assay

BTPCs were singularized with Accutase. 70,000 cells were irradiated at a dose rate of 3 Gy/min in doses ranging from 10 to 60 Gy. Irradiated cells were plated in triplicates at 20,000 cells per treatment and well of a 48-well plate. After 48 h, cells were brought to a single cell state and counted in 1:1 with Trypan Blue (Sigma-Aldrich) in a Neubauer Counting Chamber (Biochrom, Berlin, Germany). Total live cell number for each condition was normalized to non-irradiated control and expressed as a percentage difference.

### Animals

NMRI nude mice were purchased from Janvier (Le Genest-Saint-Isle, France). For experiments, 6- to 10-week-old animals were used. C57BL/6J-Tyr^c-2J^ mice were provided by the Translational Animal Research Center (TARC) of the Johannes Gutenberg University Mainz, Germany. All the mice were housed under specifically pathogen free (SPF) conditions at the TARC at a twelve-hour dark/light cycle with free access to food and water.

Animal experiments were approved by the ethics committee of the Landesuntersuchungsamt Rheinland-Pfalz and conducted according to the German Animal Protection Law §8 Abs. 1 TierSchG.

### Stereotactic injections of BTPCs

For BTPC intracranial injections, 6- to 10-week-old NMRI nude mice were used. Tumor cells were harvested by treatment with Accutase and 200,000 - 1,000,000 cells per mouse were re-suspended in 4 μl of Neurobasal-A medium naked and stored on ice until injection with a 10 μl Hamilton syringe.

30 min before surgery, experimental mice were subcutaneously injected with 400 μl of Carprofen (Rimadyl®, 4 mg/kg, Zoetis, Madison, New Jersey, USA) solution to decrease pain perception throughout and 24 h after surgery. Mice were anesthetized with intraperitoneal injection of 120 μl per 10 g body weight ketamine/xylazine mixture (40 mg/kg, 5 mg/kg) (ketamine - Ratiopharm, Ulm, Germany; xylazine - Bayer Vital, Leverkusen, Germany) and placed into the stereotactic frame (Kopf Instruments, Tujunga, California, USA). The scalp was disinfected, cut open, a burr hole drilled 2 mm lateral to the bregma into the left hemisphere and the needle introduced to a depth of 3 mm. 2 μl of cell suspension was injected at a flow rate of 400 nl/min. The injected cells were allowed to settle for 5 min before the needle was removed and the wound sewn.

### Stereotactic injections of GL261 cells

In general, 6- to 8-week-old male C57BL/6J-Tyr^c-2J^ mice were anesthetized prior to intracranial implantation by a peritoneal injection of a ketamine/xylazine mixture (120 mg ketamine and 16 mg xylazine in 10 ml of PBS) at 120 μl/10 g body weight. The anaesthetized animals were inserted into a stereotactic device and a small hole was drilled through the skull 0.5 mm anterio-posterior and 2 mm medio-lateral of the bregma. Living cells (2 x 10^5^) were slowly and carefully injected into the striatum, 3.0 mm dorso-ventral of the dura mater and the wound was closed with clamps. Subsequently, mice were observed on a daily basis for the development of glioma-specific symptoms, such as lethargy, weight loss and disheveled fur. Upon advanced progression of the tumor, they were sacrificed.

### Immunocytochemistry

Immunofluorescent stainings were performed on cells that were grown on ornithine-coated coverslips. 60,000 cells were plated 3 d prior to staining. Upon washing with PBS, cells were fixed with 4% paraformaldehyde (PFA) at RT for 5 min and permeabilized with 0.3% TritonX-100/PBS at RT for 10 min. For IFITM3 stainings, unspecific binding was blocked with 1% BSA, 0.5% NGS, 0.5% NP40 in PBS at RT for 45 min and anti-IFITM3 antibody (Abgent, San Diego, California, USA) diluted 1:200 in 1% BSA, 0.5% NP40 in PBS was applied overnight at 4°C. Cells were washed three times with PBS and incubated with secondary antibody Alexa Fluor 488-conjugated goat anti-rabbit (Invitrogen) diluted 1:1,000 in 1% BSA, 0.5% NP40 in PBS at RT. After 45 min, nuclei were counter-stained with 0.5 μg/ml DAPI (Sigma-Aldrich) in PBS at RT for 15 min. For Phalloidin stainings, unspecific binding was blocked with 5% BSA/PBS at RT for 30 min. After this blocking step, cells were once more washed with PBS and stained with 1:200 Phalloidin conjugated with Alexa Fluor 488 (Invitrogen) in 1% BSA/PBS. 1 h later nuclei were counter-stained with DAPI. For both IFITM3 and Phalloidin stainings coverslips were mounted with Fluoromount-G (SouthernBioTech, Birmingham, Alabama, USA) and images were taken using an SP5 confocal microscope (Leica, Mannheim, Germany).

### Crystal violet staining

BTPCs were dissociated with Accutase. The single cells were seeded in 1 ml of medium at a density of 2,000 cells per well previously coated with Matrigel (BD Biosciences). After two weeks, the colonies were fixed with 4% PFA at RT for 5 min and stained with 0.05% crystal violet (Sigma-Aldrich) at RT for 30 min. Cells were washed twice with tap water and 100% methanol was added to the stained cells at RT for 5 min. The optical density at 570 nm was recorded with an Infinite M200Pro Microplate Reader (Tecan, Männedorf, Switzerland).

### Immunohistochemistry

Mice were anesthetized and sacrificed by transcardial perfusion with 4% PFA. Brains were removed and incubated in 4% PFA at 4°C overnight. Subsequently, they were transferred into 30% sucrose solution and incubated at 4°C until complete infiltration. Serial free floating thick sections (40 μm) were performed. Brain sections were incubated overnight with mouse anti-claudin-5 (1:200, Invitrogen), rabbit anti-Iba-1 (1:500, Wako Chemicals, Richmond, Virginia, USA) or rabbit anti-IFITM3 (1:50, Abcam, Cambridge, Massachusetts, USA) primary antibodies followed by incubation with Alexa Fluor 568-conjugated goat anti-mouse, Alexa Fluor 647-conjugated goat anti-rabbit or Alexa Fluor 488-conjugated goat anti-rabbit (Invitrogen) secondary antibodies at a dilution of 1:1,000. Cell nuclei were counterstained with 0.5 μg/ml DAPI at RT for 5 min. Images were captured using an SP8 confocal microscope (Leica). 3D reconstruction and analysis were performed using Imaris 8 (Bitplane, Zurich, Switzerland) or ImageJ software v1.41 (National Institute of Health, Bethesda, Maryland, USA).

### RNA isolation, cDNA synthesis and qRT-PCR

RNA was isolated from cells using RNeasy mini kit (Qiagen) according to manufacturer's instructions. Concentration and purity of RNA were determined by using the Bioanalyzer 2000 automated electrophoresis system (Agilent, Santa Clara, California, USA). 1 μg of total RNA per sample was used to synthesize cDNA using random hexamer primers and avian reverse transcriptase (iScript cDNA Synthesis Kit, Bio-Rad, Hercules, California, USA) according to manufacturer's protocol. Concentration and purity of generated cDNA were determined photometrically by absorption at 260 and 280 nm wavelengths (Biophotometer, Eppendorf, Hamburg, Germany).

qRT-PCR was performed using SYBR Green fluorescein mix (Thermo Fisher Scientific). 350 ng of template cDNA and 1 pmol of gene-specific primers were used per reaction. PCR reaction and amplicon detection were performed by the iCycler real-time PCR system (CFX Connect RT-PCR System, Bio-Rad). Quantification of expression was normalized according to the relative levels of cDNA in the samples based on quantitative analysis of the house-keeping genes elongation factor 1α (Ef1α), β-actin (ACTB), glyceraldehyde-3-phosphate dehydrogenase (GAPDH) and Tyrosine 3-monooxygenase/Tryptophan 5-monooxygenase activation protein zeta (YHWAZ). Values were normalized using the CFX Manager Optical System Software (Bio-Rad). Statistical analysis was performed using the Mann-Whitney *U* - test.

### Survival analysis of GBM patients (TCGA dataset)

For the survival analysis of GBM patients we used R2: Genomics Analysis and Visualization Platform (http://r2.amc.nl) and selected the dataset Tumor Glioblastoma – TCGA – 540 - MAS5.0 - u133a with 540 samples (504 samples with clinical information). The cutoff of 656 was determined using the Kaplan scanner tool in the R2 web application. Samples were sorted according to the expression of *IFITM3* and divided into two groups on the basis of a cutoff expression value. All cutoff expression levels and the resulting groups were analyzed for survival. For each cutoff level and grouping, the log-rank significance of projected survival was calculated.

### Statistical analysis

Data were analyzed using GraphPad Prism 6 software (GraphPad Software, La Jolla, California, USA). Data are reported as means ± standard error of the mean (SEM) and differences among multiple groups were analyzed by one-way analysis of variances (ANOVA). To compare two means, the Mann-Whitney *U* - test was performed. P-values < 0.05 were considered significant. Survival curves of two groups were compared using the log rank test.
